# Temperature dependent thermal conductivity and transition mechanism in amorphous and crystalline Sb_2_Te_3_ thin films

**DOI:** 10.1038/s41598-017-14068-7

**Published:** 2017-10-23

**Authors:** Qisong Li, Jingsong Wei, Hao Sun, Kui Zhang, Zhengxing Huang, Long Zhang

**Affiliations:** 1 Laboratory for High Density Optical Storage, Shanghai Institute of Optics and Fine Mechanics, Chinese Academy of Sciences, Shanghai, 201800 PR China; 2 Key Laboratory of Materials for High Power Laser, Shanghai Institute of Optics and Fine Mechanics, Chinese Academy of Sciences, Shanghai, 201800 PR China; 3 Faculty of Electronic Information and Electrical Engineering, Dalian University of Technology, Dalian, 116024 China; 4 University of Chinese Academy of Sciences, Beijing, 100049 People’s Republic of China

## Abstract

Sb_2_Te_3_ thin films are widely used in high density optical and electronic storage, high-resolution greyscale image recording, and laser thermal lithography. Thermal conductivity and its temperature dependence are critical factors that affect the application performance of thin films. This work aims to evaluate the temperature dependence of thermal conductivity of crystalline and amorphous Sb_2_Te_3_ thin films experimentally and theoretically, and explores into the corresponding mechanism of heat transport. For crystalline Sb_2_Te_3_ thin films, the thermal conductivity was found to be 0.35 ± 0.035 W m^−1^ K^−1^ and showed weak temperature dependence. The thermal conductivity of amorphous Sb_2_Te_3_ thin films at temperatures below ~450 K is about 0.23 ± 0.023 W m^−1^K^−1^, mainly arising from the lattice as the electronic contribution is negligible; at temperatures above 450 K, the thermal conductivity experiences an abrupt increase owing to the structural change from amorphous to crystalline state. The work can provide an important guide and reference to the real applications of Sb_2_Te_3_ thin films.

## Introduction

Chalcogenide thin film materials are very useful for data storage (including optical storage and phase change random accessible memory)^1–6^ and laser thermal lithography^4–12^, where a laser or electric pulse interacts with the chalcogenide thin films and heats them to certain threshold temperature and results in a structural change between crystalline and amorphous states. For data storage, the information bits are recorded and read out according to the structural change due to contrast of reflectance or resistance between crystalline and amorphous states, accordingly^13,14^. For laser thermal lithography, the patterns can be directly inscribed on the chalcogenide thin films due to laser-induced relief structures^15^, the lithographic patterns can be also etched through acid or alkali solutions due to the selective-etching between crystalline and amorphous states^16,17^. Besides, the chalcogenide phase change materials have been explored new functions, such as optically photonic devices, optical mask layer in reducing the spot and lithographic width^18–23^, and thermoelectric devices. In these chalcogenide phase change materials, Ge-Sb-Te family^24,25^, such as Ge_2_Sb_2_Te_5_^26^, Ge_1_Sb_2_Te_4_^27^, and Ge_1_Sb_4_Te_7_^28^, are the most popular chalcogenide phase change materials in actual applications. The typical Ge_2_Sb_2_Te_5_ thin films have multi-functions^29^, such as super-resolution mask^30^, phase-selective florescence^31^ and image lithography^32^.

Actually, the basic elements of the Ge-Sb-Te family are GeTe and Sb_2_Te_3_^33,34^. The Sb_2_Te_3_ is critical for the formation and performances of Ge-Sb-Te alloys. In real application of Ge-Sb-Te alloys, whether data storage or laser thermal lithography and other aspects, the physical essence originates from the thermally-induced structural transformation, such as crystallization, amorphization, and melting. In the process of thermally-induced structural transformation, the temperature-dependent thermal conductivity of basic element of Sb_2_Te_3_ in Ge-Sb-Te alloys is one of the critical factors that determine the performances of related devices. More importantly, among chalcogenides, Sb_2_Te_3_ itself is also very useful in lots of aspects due to thermally-induced structural transformation, such as grayscale and pattern lithography^35^, thermoelectricity^36,37^, and optical nonlinearity-induced super-resolution and nano-optical information storage^38^, and topological insulators^39,40^. These applications necessitate a study of the temperature-dependent thermal properties of Sb_2_Te_3_ materials via experimental methods and accordingly, the mechanism should also be explored.

In past years, several reports were presented on the thermal properties of Sb_2_Te_3_ materials. For example, Lan *et al*. reported the thermal conductivity of solid Sb_2_Te_3_ at temperatures above room temperature^41^. Masashi *et al*. measured the thermal conductivities of crystalline Sb_2_Te, Sb_2_Te_3_, SbTe_9_, Sb, and Te thin films at room temperature^42^. Wang *et al*. studied the thermal conductivity of nitrogen-doped Sb_2_Te_3_ thin films as a function of temperature^43^.

However, the temperature dependence of thermal conductivity and its mechanism in case of Sb_2_Te_3_ thin films, including both its crystalline and amorphous states, and the change of thermal conductivity in the process of structural transformation from crystalline to amorphous states still needs to be explored. This work aims to experimentally obtain the thermal conductivities, electric conductivities and Hall coefficient of amorphous and crystalline Sb_2_Te_3_ thin films, especially as a function of temperature. Furthermore, the physical mechanism and the contributions from lattice and electron thermal conductivities in the process of thermally-induced structural transformation have also been studied.

## Experimental Details

### Sample Preparation

**Figure 1:**
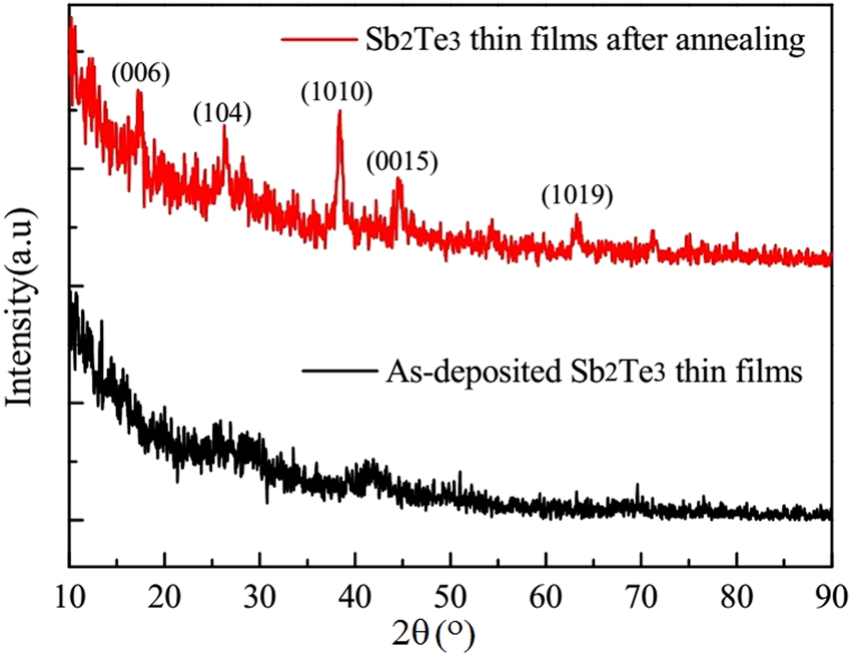
The XRD results of Sb_2_Te_3_ thin films.

Amorphous Sb_2_Te_3_ thin films with various thicknesses were directly deposited on p-type Si (100) wafers at room temperature by radio-frequency (RF) magnetron sputtering using a single alloy target of Sb_2_Te_3_. The background pressure was approximately 5 × 10^−4^ Pa and the sputtering pressure of 0.5 Pa was obtained by filling Ar gas in the chamber. The sputtering power was set at 40 W and the deposition rate was close to 1 nm per second. Five samples with thicknesses from 46.5 nm to 180 nm have been prepared. The crystalline Sb_2_Te_3_ thin films were obtained by annealing at temperature 520 K under a vacuum atmosphere for 30 min. The thicknesses of the thin films before and after annealing are considered to be unchanged. The structures of the Sb_2_Te_3_ thin films were measured by X-Ray diffraction (XRD) (Fig. 1). XRD results indicate that the as-deposited samples were amorphous while the annealed samples were crystalline.

### Temperature-dependent thermal conductivity

The variable-temperature thermal conductivity measurements are carried out by using the transient thermoreflectance technique (TTR)^44,45^. The main advantage of this technique is that it is a non-contacting and non-destructive optical method, both for heating a sample under test and for probing the variations of its surface temperature. Furthermore, both sample preparation and the experimental setup are simple to execute.

### Establishment of TTR measurement system

**Figure 2:**
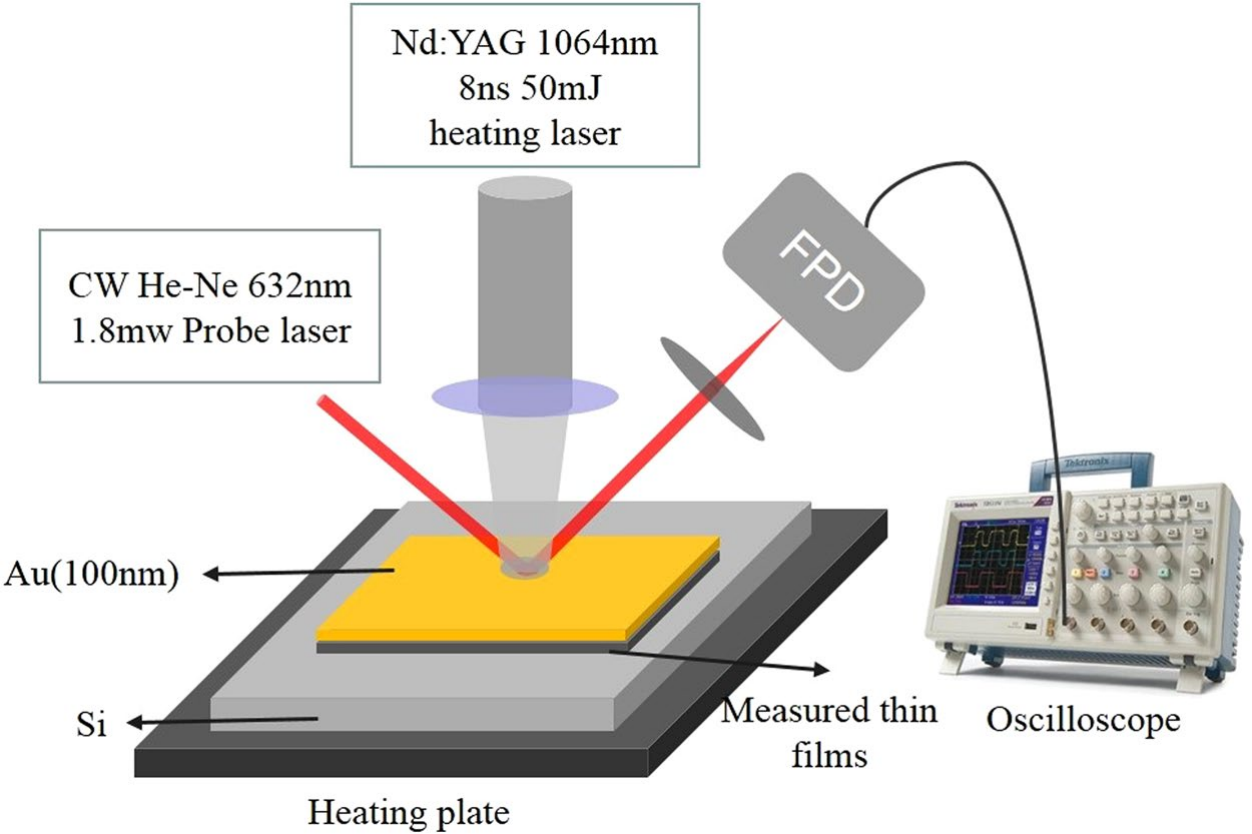
Illustration of the transient thermoreflectance technique (FPD: fast photodetector).

The principle of the TTR setup is shown in Fig. 2. The heating laser is an ultra-compact Nd:YAG pulsed laser with a wavelength of 1064 nm, pulse width of 8 ns, single pulse energy of 50 mJ, and the repetition rate as 1–20 Hz. The probing laser is a continuous He–Ne laser with a wavelength of 632 nm and power 1.8 mW. The heating laser is used to irradiate the surface of the metal layer, which induces an increase in its temperature. Subsequently, the surface temperature decreases over time owing to the heat diffusing to the thin film layer. Since the reflectivity of metals is sensitive to their temperatures and approximately proportional to the temperatures in a wide but limited temperature range^46^, the normalized temperature profile can be obtained from the normalized reflected light intensity. The reflected probe light from the heated metal surface is guided to a fast photodetector and the signal of the photodetector is collected and averaged by an oscilloscope.

### Model fitting of thermal conductivity

In the fitting model, the transmission-line theory is utilized to establish the theoretical model from the 1-D heat conduction equation and the genetic algorithms (GAs) is used to obtain the fitting result^47,48^. Considering the thickness of thin films as a thermal resistance layer, a theory model for the double-layer system is used to obtain the thermal conductivity of films through the fitting curve. The temperature expression for the double-layer model in Laplace domain is^44^:

1 $${\rm{T}}(s)=\frac{1}{{e}_{1}\sqrt{s}}\frac{{\cos }^{-1}{\mu }_{1}\sqrt{s}+{e}_{21}{\sin }^{-1}{\mu }_{1}\sqrt{s}+{r}_{th}{e}_{2}\sqrt{s}{\cos }^{-1}{\mu }_{1}\sqrt{s}}{{\sin }^{-1}{\mu }_{1}\sqrt{s}+{e}_{21}{\cos }^{-1}{\mu }_{1}\sqrt{s}+{r}_{th}{e}_{2}\sqrt{s}{\sin }^{-1}{\mu }_{1}\sqrt{s}}$$

where ${e}_{i}=\sqrt{{\rho }_{i}{c}_{i}{k}_{i}}$, ${e}_{i,j}=\frac{{e}_{i}}{{e}_{j}}$, ${\mu }_{i}={d}_{i}\sqrt{{\rho }_{i}{c}_{i}}/\sqrt{{k}_{i}}$, i = 1, 2 represent the metal layer and the substrate, respectively. The terms *k*, *ρ*, *c*, and *d* refer to the thermal conductivity, density, specific heat, and the thickness of thin film, respectively. ${r}_{th}$ is the total thermal resistance including the thermal resistance of the sample and the interfacial thermal resistance *r*_c_ between the sample and the metal layer or the substrate. The interfacial thermal resistance can be extracted from the total thermal resistance under different thickness using *r*_th_ = *d*/*k* + *r*_c_, if thermal conductivity is unity for thin films of different thicknesses. Owing to the complex temperature expression in the frequency domain, it is impossible to directly obtain the temperature-time expression in the time domain. The inverse Laplace transform using the Stehfest numerical method is used^48^. The mathematical expression is:

2 $${\rm{f}}(t)=\frac{ln2}{t}\sum _{1}^{N}{A}_{n}F(\frac{nln2}{t})$$

and

$${A}_{n}={(-1)}^{n+N/2}\sum _{j=[(n+1)/2]}^{min(n,N/2)}\frac{{j}^{N/2}(2j)!}{(N/2-j)!j!(j-1)!(n-j)!(2j-n)!}$$

where $F(s)$ is the Laplace transform formula of ${\rm{f}}(t)$ and the bracket of j means an integer. In order to fit the experimental data with the theoretical model, the bulk density and the specific heat (2.49 × 10^6^ J m^−3^ K^−1^) of the metal layer and the bulk thermal conductivity (148 W m^−1^ K^−1^) of the substrate at room temperature are used. In our measurement, the metal layer is only used as light absorption layer. The temperature decay curve directly embodies the thermal diffusion process of Sb_2_Te_3_ thin film sample. In the fitting, the thermal conductivity of 237 W m^−1^ K^−1^ for Au thin film is used^44^. Thus, one only needs to fit the *r*_*th*_ to extract the thermal conductivity values of Sb_2_Te_3_ thin films by using the GA. The objective function used for the GA is written as follows^49^:

3 $${f}_{0}=\sum _{i=1}^{{N}_{r}}{({T}_{e}(i)-{T}_{t}(i))}^{2}\,$$

where ${T}_{e}(i)$ and ${T}_{t}(i)$ are the experimental and theoretical normalized temperatures, respectively, and N_r_ is the number of measurements recorded over time. Finally, the value of the thermal conductivity can be extracted by minimizing the objective function.

## Results and Discussion

### The results of thermal conductivity measurement

In practical applications, the thickness of Sb_2_Te_3_ thin films is usually changed according to different requirements and the thermal conductivity also changes with temperature. Thus, the dependence of thermal conductivity on both thin film thickness and temperature were measured using the setup shown in Fig. 2. In the thermal conductivity measurements, in order to enhance the heat absorption and become able to detect the variation of temperature, a gold layer of 120 nm thickness was deposited on the top of the Sb_2_Te_3_ thin films.

### Thickness-dependent thermal conductivity

**Figure 3:**
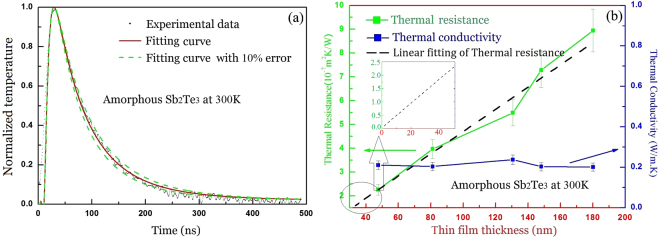
(**a**) The temperature delay curve of amorphous Sb_2_Te_3_ thin films with the thickness 46.5 nm. The experimental and fitting curves of temperature change with time (the value of thermal conductivity is 0.23 W m^−1^ K^−1^. (**b**) The dependence of thermal resistance and conductivity on the thickness of the thin film and the inset is a local amplification, the error bar is set by ±10%.

Figure 3a illustrates the experimental and fitting curves of the temperature change with time for the 46.5 nm thin film. Figure 3b shows the obtained thermal resistances and the thermal conductivities of the thin films. It can be seen that the total thermal resistance increases linearly with the increasing thickness and the interfacial thermal resistance is evaluated by linearly fitting the *r*_th_-*d* relation. The lumped interfacial thermal resistance including both Au/thin-film and thin-film/Si-substrate interfaces for amorphous thin film is ~0.04 × 10^−7^ m^2^ K W^−1^, which can be obtained from the intercept value of the linear fitting in the inset of Fig. 3b. By theoretical fitting, the thermal conductivity in Fig. 3a is found to be *k* = 0.23 ± 0.023 W m^−1^ K^−1^, and the uncertainty of this value is evaluated as 10%, which is mainly from the thickness measurement of the thin films. In order to consider the error in experimental data, fitting curves with ±10% error are also shown.

**Figure 4:**
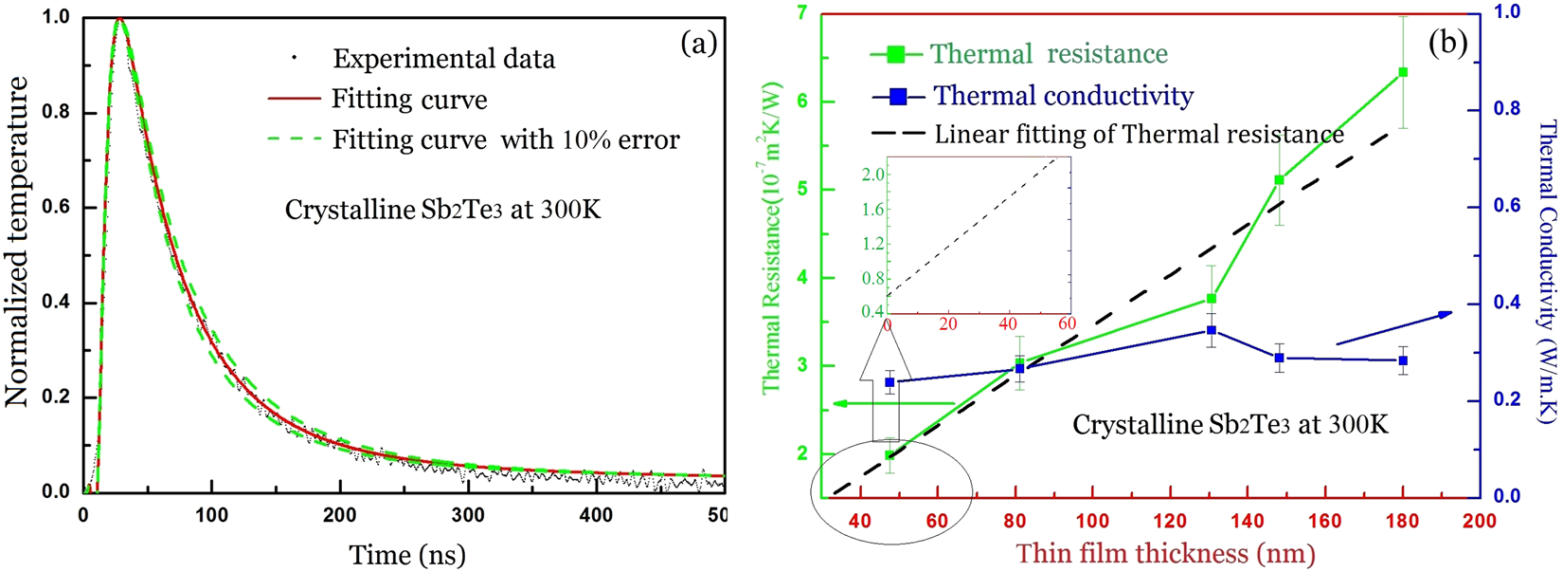
(**a**) The temperature delay curve of crystalline Sb_2_Te_3_ thin films with the thickness 47.4 nm. The experimental and fitting curves of temperature decay with time (the value of thermal conductivity is 0.31 W m^−1^ K^−1^). (**b**) The dependence of thermal resistivity and conductivity on the thickness of the thin film and the inset is a local amplification, the error bar is set by ±10%.

The thermal conductivity of crystalline Sb_2_Te_3_ thin films can be obtained from the normalized experimental data and fitting curves, as shown in Fig. 4. Similar to Fig. 3b, the thermal resistance shows an almost linear increase with the thin film thickness. Figure 4a illustrates the experimental and fitting curves of temperature change with time for a crystalline Sb_2_Te_3_ thin film with a thickness of 47.4 nm. As compared with amorphous thin films, the temperature decay time of crystalline thin films is short, which implies that the thermal diffusion capacity of the crystalline is larger than that of the amorphous state. The lumped interfacial thermal resistance including both Au/thin-film and thin-film/Si-substrate interfaces for crystalline thin films is ~0.6 × 10^−7^ m^2^ K W^−1^, which can be obtained from the intercept value of the linear fitting in the inset of Fig. 4b. By theoretical fitting, the thermal conductivity in Fig. 4a is found to be *k* = 0.35 ± 0.035 W m^−1^ K^−1^. Comparison of Figs 3 and 4 indicates that the thermal conductivity of Sb_2_Te_3_ thin films, whether the crystalline or amorphous state, does not change with thin film thickness. It is noted that the measured values of thermal resistance for amorphous and crystalline samples with ~130 nm thickness deviate from linear assumption, which is owning to the sample preparation and the measurement error of sample thickness and the measurement error on sample thicknesses is ±7% for the amorphous and crystalline thin films. Furthermore, the thermal conductivity of crystalline Sb_2_Te_3_ thin films is larger than that of amorphous state.

### Temperature dependence of thermal conductivity

**Figure 5:**
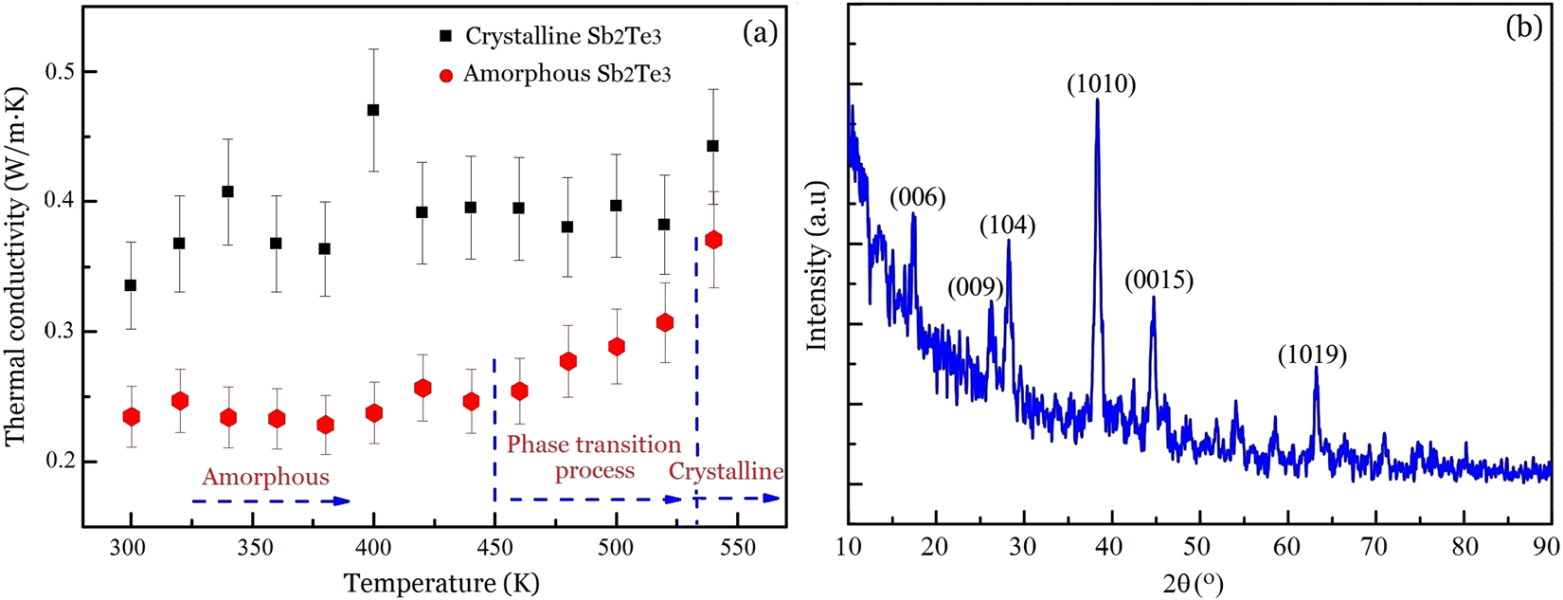
The measured thermal conductivity of the Sb_2_Te_3_ thin film with a thickness of 180 nm by the TTR method. (**a**) Thermal conductivity values of amorphous and crystalline Sb_2_Te_3_ thin films as a function of temperature, the error bar is set by ±10%. (**b**) XRD analysis of the sample after the variable temperature thermal conductivity measurement.

The thermal conductivities of the crystalline and amorphous thin film with the thickness of 180 nm were measured as a function of temperature as shown in Fig. 5a. For crystalline Sb_2_Te_3_ thin films, the thermal conductivity displays very little change between 0.35 ± 0.035 W m^−1^ K^−1^ and $0.38\pm 0.038$ W m^−1^ K^−1^ in the temperature range from 300 K to 540 K. In the case of amorphous Sb_2_Te_3_ thin films, the thermal conductivity is about 0.23 ± 0.023 W m^−1^ K^−1^ when the temperature is below 450 K. Increasing the temperature from 450 K to 540 K increases the thermal conductivity from 0.25 ± 0.025 W m^−1^ K^−1^ to $0.37\pm 0.037$ W m^−1^ K^−1^, which can be regarded as a result of the phase transition from the amorphous to crystalline state and accord with the report in literature^50^. At 540 K, the thermal conductivity value is nearly equal to that of crystalline Sb_2_Te_3_ thin films. This indicates that the amorphous Sb_2_Te_3_ thin films were completely crystallized by the variable temperature TTR measurement. This result is further confirmed by XRD analysis, as shown in Fig. 5b, where the strong crystalline peaks occurs and the crystalline peaks are almost the same as the crystalline peaks of the blue XRD curve in Fig. 1. That is, the amorphous Sb_2_Te_3_ thin films are transformed into the hexagonal crystalline state in the process of variable-temperature TTR measurement. Thus, this measurement may be considered as a phase transition (crystallization) process.

### Mechanism of temperature-dependent thermal conductivity

Thermal conductivity of semiconductor materials is mainly comprised of electronic thermal conductivity and lattice thermal conductivity. The mechanism of temperature-dependent thermal conductivity can also be determined by considering the contributions from electrons and lattices.

### Electron contribution to total thermal conductivity

**Figure 6:**
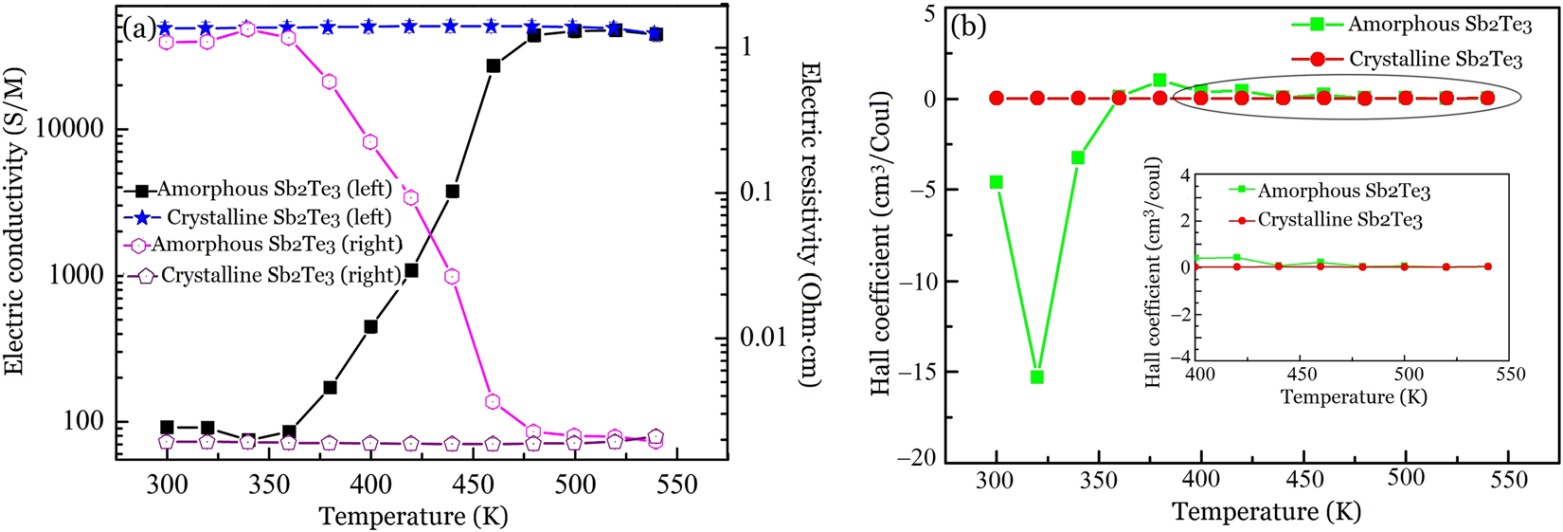
(**a**) Electrical conductivity and resistivity measurements and (**b**) Hall coefficient of amorphous and crystalline Sb_2_Te_3_ thin films as a function of temperature.

In order to explore the thermal conduction mechanism of the phase transition process, the electric resistivity, conductivity, and Hall Coefficient of the amorphous and crystalline Sb_2_Te_3_ thin films were measured from 300 K to 540 K by using the MMR Hall and Van der Pauw measurement system^51^. Figure 6 presents the experimental results, where the sample thickness is 180 nm and the measurement error is ±10%. Figure 6a illustrates the temperature dependence of electric resistivity *ρ* of crystalline and as-deposited samples. The value of *ρ* for crystalline Sb_2_Te_3_ thin films was determined to be $0.001\pm 0.0001$ Ohm.cm and it was independent of the temperature. For as-deposited Sb_2_Te_3_ thin films, *ρ* was approximately $1.2\pm 0.12$ Ohm.cm at below 360 K. The *ρ* value of crystalline films was three orders of magnitude lower than the value of the amorphous films. The value of *ρ* for amorphous films abruptly decreased from $1.2\pm 0.12$ to $0.001\pm 0.0001$ Ohm.cm on increasing the temperature from 360 K to 500 K. According to refs^52,53^, the change of electrical resistivity is regarded as the phase transition process and the fall in electrical resistivity is due to the structural change from the amorphous to the crystalline. Thus, one can see that it is also indicative of the phase transition from the amorphous to crystalline state above ~360 K. The *ρ* value remains unchanged above 500 K because the crystallization is completed above this temperature. The electric conductivity is calculated through σ$\,\propto \,1/\rho$, and as shown in Fig. 6a, σ is below 100 ${\rm{S}}/{\rm{M}}$ for the amorphous state and does not change with temperature below ~360 K, and then abruptly increases from $\mathrm{below}\,\,100\,{\rm{S}}/{\rm{M}}$ to $(5\,\pm \,0.5)\,\times \,{10}^{4}\,{\rm{S}}/{\rm{M}}$ as the temperature increases from 360 K to 500 K. and then remains at $(5\,\pm \,0.5)\,\times \,{10}^{4}\,{\rm{S}}/{\rm{M}}$ and unchanged above 500 K due to the complete crystallization. The value of σ for the crystalline state is three orders higher than that of the amorphous state.

In order to better understand the mechanism of electrical conduction, the Hall coefficient was measured as a function of temperature, as shown in Fig. 6b. For crystalline Sb_2_Te_3_ thin films, the Hall coefficient was always a positive value, which indicates that holes are the main charge carriers. For amorphous Sb_2_Te_3_ thin films, the Hall coefficient was found to be negative for temperatures from 300 K~340 K and close to zero at 360 K, which implies that electrons were the primary carriers. At temperatures above 375 K, the Hall coefficient became positive, which means that the holes become the charge carriers. The transition of the carrier type from electrons to holes results from the structural change in Sb_2_Te_3_ from amorphous to crystalline at 375 K. In general, a larger value of the Hall coefficient implies a large resistivity of the material. Thus, it may be concluded that the resistivity of amorphous Sb_2_Te_3_ thin films is larger than that of the crystalline thin films.

Based on Wiedemann–Franz (W-F) law^54^, the electronic thermal conductivity for both metals and semiconductors is related to their electric conductivity. The W-F law is written as follows:

4 $${\kappa }_{e}=LT\sigma =\frac{{\pi }^{2}}{3}{(\frac{{k}_{B}}{e})}^{2}\sigma T$$

where ${\kappa }_{e}$ is the electronic thermal conductivity (W m^−1^ K^−1^), *σ* is the electric conductivity (S m^−1^), T is the temperature, the Lorenz number L = $\frac{{\pi }^{2}}{3}{(\frac{{k}_{B}}{e})}^{2}$, and ${k}_{B}$ is the Boltzmann constant. The Sb_2_Te_3_ thin films are one of compound semiconductor alloys, and their electric conductivity measurements were conducted at temperatures that were above the room temperature, and thus above the Debye temperature of 162 K. Thus, the Sb_2_Te_3_ thin film can be considered as non-degenerate semiconductors. The Lorenz number can be regarded as a constant value of 1.48 × 10^−8^ W Ω K^−2^ for non-degenerate semiconductors in the temperature range from 100 K to 800 K^55^.

**Figure 7:**
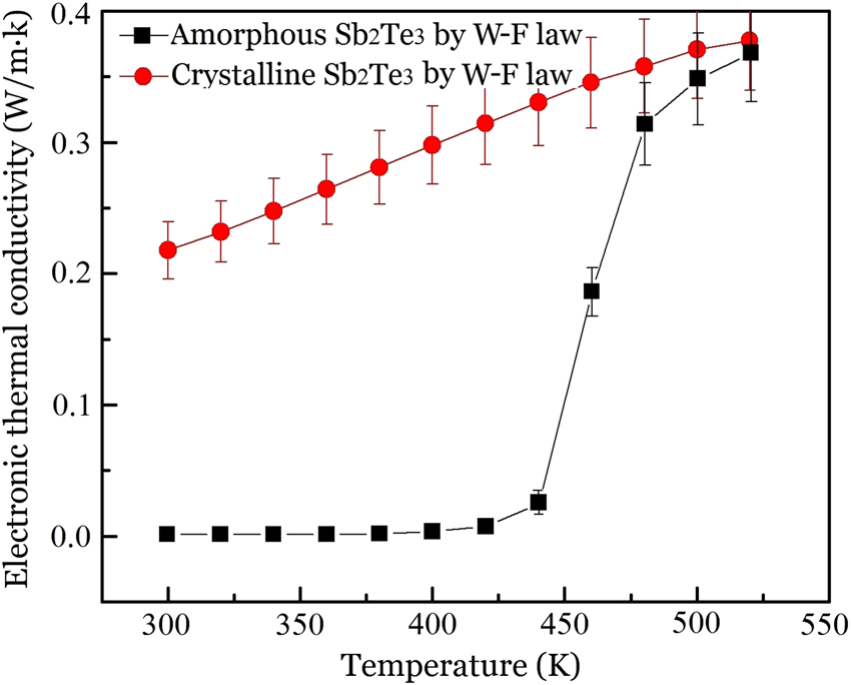
Electronic thermal conductivity values of amorphous and crystalline Sb_2_Te_3_ thin films as a function of temperature, which are calculated by W-F law and electrical conductivity data of Fig. 6(a), the error bar is set by ±10%.

Figure 7 gives the electronic thermal conductivity of Sb_2_Te_3_ thin film calculated from the electric conductivity results in Fig. 6a. For crystalline Sb_2_Te_3_ thin films, the electronic thermal conductivity increases linearly from (0.22 ± 0.022) W m^−1^ K^−1^ to (0.37 ± 0.037) W m^−1^ K^−1^ as the temperature increases from 300 K to 520 K. For amorphous Sb_2_Te_3_ thin films, the electronic thermal conductivity is very small and can be neglected at below 450 K. At above 450 K, the electronic thermal conductivity sharply increases and almost becomes equal to the value of crystalline Sb_2_Te_3_ thin films at 520 K due to crystallization effect. Compared with the results shown in Fig. 5 for amorphous Sb_2_Te_3_ thin films, the contribution of electronic thermal conductivity to total thermal conductivity can be ignored at below 450 K. However, for crystalline Sb_2_Te_3_ thin films, the electronic thermal conductivity presents large contribution to the total thermal conductivity.

It should be noted that, in Fig. 5a, the total thermal conductivity of amorphous sample (red symbol) experiences an abrupt change in the temperature range from 450 K to 520 K, resulting from the structural change from amorphous to crystalline state. Comparing Figs 5a and 7, one can note that, during the structural change of amorphous sample, the total thermal conductivity is close to the electronic thermal conductivity in the range of error. For example, the total thermal conductivity value is about $0.31\pm 0.031\,\,$W m^−1^ K^−1^ at 520 K, which is very close to the calculated electronic thermal conductivity value of about $0.35\pm 0.035\,\,$W m^−1^ K^−1^. Generally speaking, the calculated electronic thermal conductivity should be a little smaller than the total thermal conductivity due to the contribution from phonon part. However, there appears to be a little contradiction between the calculated electronic thermal conductivity and the total thermal conductivity. The little discrepancy may be because the W-F model is an ideal model, only suitable for ideal materials, and not completely suitable for the materials with defects and mixture/compound materials, as pointed out in refs^55,56^. In the temperature range from about 450 K to 520 K, on the one hand, the amorphous sample is actually a mixture/compound material of amorphous and crystalline states, and that is, the sample is in the transition state from amorphous state to crystalline. On the other hand, there are also some defects with the concentration 28% of the total volume in the crystalline part, the defects can be considered as cavities, which is a discrete phase in the matrix.

Actually, the discrepancies have been reported in other literatures. For example, in ref.^55^, the electronic thermal conductivities calculated by W-F law are higher than the total thermal conductivities below 500 K. The authors thought that the use of the theoretical value of the Lorentz number is not suitable for the calculation of all alloys, since its absolute value changes depending upon the type of material and the temperature. In ref.^56^, the electronic thermal conductivities are also higher than the measured total thermal conductivities below 600 K, which maybe result from the sample itself and the different measurement methods. In our work, to give a reference for the thermal conductivity contribution of electronic part, thus, we are reluctant to use the W-F model to roughly estimate the electronic thermal conductivity. Therefore, a little discrepancy between the calculated electronic thermal conductivity and the total thermal conductivity occurs. That is, although the electronic component could not be precisely estimated, one can still deduce that, in the transition state from amorphous to crystalline state of Sb_2_Te_3_ thin films, the electronic thermal conductivity dominates the total thermal conduction, and the lattice component can be neglected.

### Lattice contribution to total thermal conductivity

The lattice thermal conductivity is attributed to the heat conduction of the phonon gas enclosed in the crystal, and can be obtained from the kinetic theory^57^:

5 $${\kappa }_{\iota }=\frac{1}{3}{C}_{V}\nu {l}_{mfp}$$

where ${C}_{V}$, $\nu$, and ${l}_{mfp}$ refer to the heat capacity at constant volume, average phonon velocity, and mean free path of phonons, respectively. The temperature dependence of lattice thermal conductivity is normally treated within the Debye approximation model. In this model, $\nu \,\,$ is assumed to be independent of temperature and the relationship between ${\kappa }_{\iota }$ and temperature is determined by evaluating the variation of ${C}_{V}$ and $\,{l}_{mfp}$ with temperature. The average energy of lattice vibrations can be expressed as follows:

6 $${\rm{E}}=\frac{qN}{{{\omega }_{D}}^{3}}{\int }_{0}^{{\omega }_{D}}(\frac{1}{2}\hslash \omega +\frac{\hslash \omega }{{e}^{\hslash \omega /{k}_{B}T}-1}){\omega }^{2}d\omega $$

where *q*, *T*, *N*, $\hslash$, ${k}_{B}$, and $\omega$ refer to the wave vector, temperature (in Kelvin), Avogadro constant, Planck constant, Boltzmann constant, and frequency of the lattice wave, respectively. ${\omega }_{D}=\nu {(\frac{6{\pi }^{2}N}{{V}_{m}})}^{1/3}\,$is the upper frequency limit set in the Debye model with the molar volume ${V}_{m}$.

The lattice heat capacity ${C}_{V}$ can be calculated as:

7 $${C}_{V}={(\frac{\partial E}{\partial T})}_{V}=qN{k}_{B}{(\frac{T}{{{\rm{\Theta }}}_{D}})}^{3}{\int }_{0}^{{{\rm{\Theta }}}_{D}/T}\frac{{x}^{4}{e}^{x}}{{({e}^{x}-1)}^{2}}dx$$

In this formula, ${{\rm{\Theta }}}_{D}=\hslash {\omega }_{D}/{k}_{B}$ is the Debye temperature. At low temperatures,${\rm{T}}\ll {{\rm{\Theta }}}_{D}$, the expression ${C}_{V}$ can be approximated as:

8 $${C}_{V}=\frac{12{e}^{4}}{5}N{k}_{B}{(\frac{T}{{{\rm{\Theta }}}_{D}})}^{3}$$

Formula (8) is called as Debye T^3^ law. At high temperatures,${\rm{T}} > {\Theta }_{D}$, Formula (7) can be written as:

9 $${C}_{V}=3N{k}_{B}$$

Formula (9) indicates that the lattice heat capacity ${C}_{V}$ is independent of temperature. This is also called as the Dulong-Petit law.

**Figure 8:**
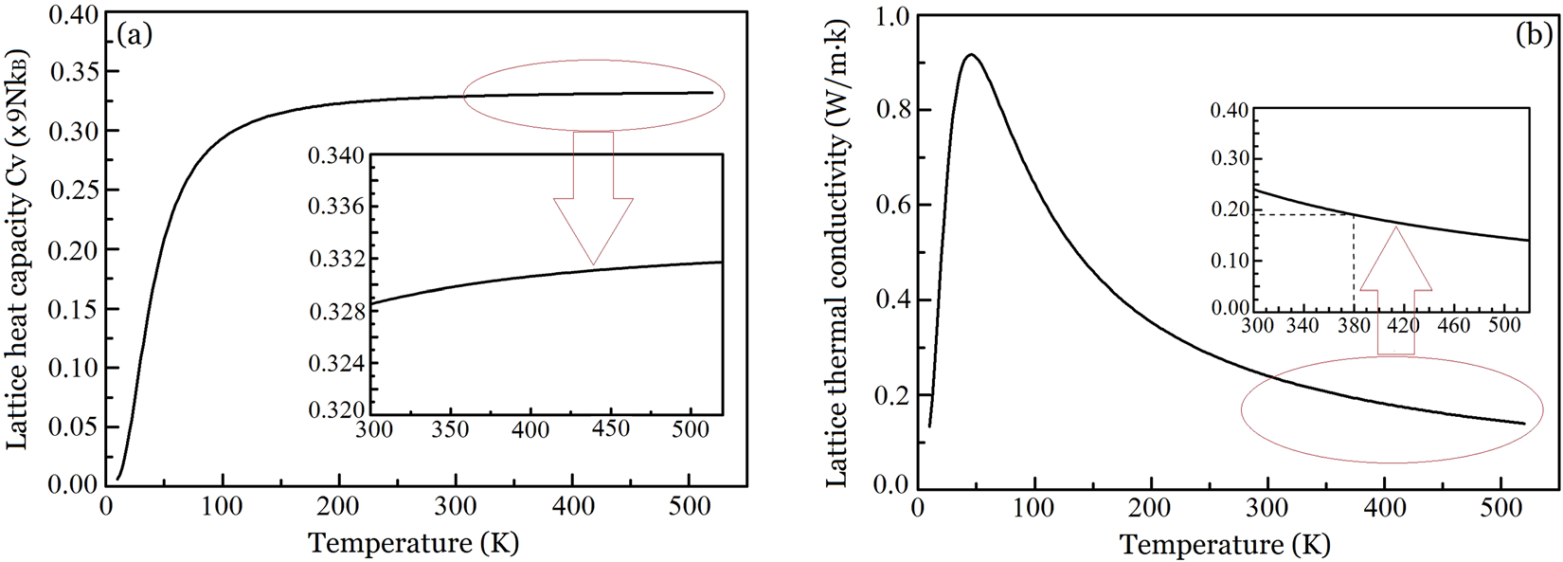
Thermal properties of Sb_2_Te_3_ thin films as a function of temperature, where the insets are amplification. (**a**) Lattice heat capacity calculated from the Debye model, (**b**) thermal conductivity calculated from the Debye model and kinetic theory.

Given that the speed of sound traveling speed in Sb_2_Te_3_ is 2300 m s^−1^ ^58^, the Debye temperature ${{\rm{\Theta }}}_{D}$ is calculated to be 162 K. Figure 8a shows the lattice heat capacity ${C}_{V}$ as a function of temperature. It is evident that in the temperature range from 300 K to 520 K, the lattice heat capacity ${C}_{V}$ increase very little with the rise in temperature (Fig. 8a, inset).

The mean free path of phonons is determined by the phonon collisions, impurities, and defects. At T > ${{\rm{\Theta }}}_{D}$, the average number of phonons ${\rm{n}}(\omega )$ is written as^59^:

10 $${\rm{n}}(\omega )=\frac{1}{\exp (\hslash \omega /{k}_{B}T)-1}\approx \frac{{k}_{B}T}{\hslash \omega }$$

Formula (10) indicates that the phonon number is positively proportional to temperature. It is known that the mean free path of phonons is inversely proportional to phonon number, thus the mean free path of phonons is also inversely proportional to temperature, i.e. ${l}_{mfp}\approx A/T$, where A is the constant. Based on Eq. (7), the lattice thermal conductivity is also inversely proportional to temperature and written as:

11 $${\kappa }_{\iota }=\frac{1}{3}{C}_{V}\nu {l}_{mfp}\approx \frac{1}{3}qN{k}_{B}\nu \frac{A}{T}{(\frac{T}{{{\Theta }}_{D}})}^{3}{\int }_{0}^{{\Theta }_{D}/T}\frac{{x}^{4}{e}^{x}}{{({e}^{x}-1)}^{2}}dx$$

According to the known parameters of Sb_2_Te_3_ at room temperature, namely, ${C}_{v}$ = 206 J kg^−1^ K^−1^ ^60^, $\nu$ = 2300 m s^−1^ ^58^, and ${l}_{mfp}$ = 2.2 Å for thin films^61^, the lattice thermal conductivity is theoretically calculated to be ~0.24 W m^−1^ K^−1^, which is consistent with the reported values^61,62^. Therefore, the coefficient of the lattice thermal conductivity equation can be fixed. The relation between lattice thermal conductivity and temperature can be obtained by fitting the Eq. (11).

Figure 8b shows the lattice thermal conductivity of the crystalline thin films as a function of temperature. The inset is an amplification of the temperature range from 300 K to 520 K, and it can be seen that the lattice thermal conductivity is 0.24 W m^−1^ K^−1^ at 300 K, and then gradually decreases to 0.14 W m^−1^ K^−1^ at 520 K. By comparing, one can see that the lattice thermal conductivities of crystalline state are approximately to those of amorphous state calculated from Figs 5a and 7. This may be because the pair-correlation functions, phonon density of states and electronic density of states in amorphous Sb_2_Te_3_ are approximately to those of crystalline Sb_2_Te_3_^63^. For crystalline Sb_2_Te_3_ thin films, both the lattice thermal conductivity and electronic thermal conductivity make important contributions to the total thermal conductivity. The temperature coefficients of lattice and electronic thermal conductivity are positive and negative, respectively, which leads to the weak temperature dependence of total thermal conductivity of crystalline Sb_2_Te_3_ thin films.

## Conclusion

In this work, the changes in thermal conductivity of crystalline and amorphous Sb_2_Te_3_ thin films have been investigated both experimentally and theoretically. For crystalline Sb_2_Te_3_ thin films, the thermal conductivity was found to be $0.35\pm 0.035$ W m^−1^ K^−1^ and it slowly increases with temperature rising. Both the lattice and electronic thermal conductivities make important contributions to the total thermal conductivity. The temperature coefficients of lattice and electronic thermal conductivity were positive and negative, respectively. This is the origin of the weak temperature dependence of total thermal conductivity in the case of crystalline Sb_2_Te_3_ thin films. At temperatures below ~450 K, the thermal conductivity of amorphous Sb_2_Te_3_ thin films was found to be 0.23 ± 0.023 W m^−1^ K^−1^ and showed little temperature dependence, with the main contribution arising from the lattice and that of the electrons being ignorable. At temperatures above 450 K, the thermal conductivity experienced an abrupt increase as a result of the structural change from amorphous to crystalline state.
